# TROG 15.03 phase II clinical trial of Focal Ablative STereotactic Radiosurgery for Cancers of the Kidney - FASTRACK II

**DOI:** 10.1186/s12885-018-4916-2

**Published:** 2018-10-23

**Authors:** Shankar Siva, Brent Chesson, Mathias Bressel, David Pryor, Braden Higgs, Hayley M. Reynolds, Nicholas Hardcastle, Rebecca Montgomery, Ben Vanneste, Vincent Khoo, Jeremy Ruben, Eddie Lau, Michael S. Hofman, Richard De Abreu Lourenco, Swetha Sridharan, Nicholas R. Brook, Jarad Martin, Nathan Lawrentschuk, Tomas Kron, Farshad Foroudi

**Affiliations:** 1 Peter MacCallum Cancer Centre, 305 Grattan Street Melbourne, Melbourne, 3000 Australia; 2 University of Melbourne, Royal Parade, Parkville, 8006 Australia; 3 Princess Alexandra Hospital, Brisbane, QLD Australia; 4 University of Adelaide, Royal Adelaide Hospital, Adelaide, South Australia Australia; 5 Trans Tasman Radiation Oncology Group (TROG), Waratah, Australia; 6 MAASTRO Clinic, Maastricht, The Netherlands; 7 Royal Marsden Hospital, London, UK; 8 Alfred Health and Monash University, 55 Commercial Rd, Melbourne, 3004 Australia; 9 Austin Health, Heidelberg, 3084 Australia; 10 Centre for Health Economics Research and Evaluation, University of Technology Sydney, Sydney, Australia; 11 Calvary Mater Newcastle, Newcastle, NSW Australia; 12 Radiation Oncology, Austin Health, Heidelberg, Australia

**Keywords:** SBRT, SABR, RCC, Metastases, Kidney, Adrenal, Ablation, Nephrectomy

## Abstract

**Background:**

Stereotactic ablative body radiotherapy (SABR) is a non-invasive alternative to surgery to control primary renal cell cancer (RCC) in patients that are medically inoperable or at high-risk of post-surgical dialysis. The objective of the FASTRACK II clinical trial is to investigate the efficacy of SABR for primary RCC.

**Methods:**

FASTRACK II is a single arm, multi-institutional phase II study. Seventy patients will be recruited over 3 years and followed for a total of 5 years. Eligible patients must have a biopsy confirmed diagnosis of primary RCC with a single lesion within a kidney, have ECOG performance ≤2 and be medically inoperable, high risk or decline surgery. Radiotherapy treatment planning is undertaken using four dimensional CT scanning to incorporate the impact of respiratory motion. Treatment must be delivered using a conformal or intensity modulated technique including IMRT, VMAT, Cyberknife or Tomotherapy. The trial includes two alternate fractionation schedules based on tumour size: for tumours ≤4 cm in maximum diameter a single fraction of 26Gy is delivered; and for tumours > 4 cm in maximum diameter 42Gy in three fractions is delivered. The primary outcome of the study is to estimate the efficacy of SABR for primary RCC. Secondary objectives include estimating tolerability, characterising overall survival and cancer specific survival, estimating the distant failure rate, describing toxicity and renal function changes after SABR, and assessment of cost-effectiveness of SABR compared with current therapies.

**Discussion:**

The present study design allows for multicentre prospective validation of the efficacy of SABR for primary RCC that has been observed from prior single institutional and retrospective series. The study also allows assessment of treatment related toxicity, overall survival, cancer specific survival, freedom from distant failure and renal function post therapy.

**Trial registration:**

Clinicaltrials.gov NCT02613819, registered Nov 25th 2015.

## Background

### Renal cell carcinoma and SABR

Primary renal cell carcinoma (RCC) is the 9th most common cancer in Australia [1] and 8th most common worldwide [2]. In Australia there has been an 88% increase in the age-standardised incidence of RCC in the period from 1982 to 2014 [1]. The standard of care for fit patients with localised kidney cancer is surgical resection of the kidney (nephrectomy). However, patients undergoing partial or total nephrectomy for renal cancer experience post-operative nephron loss, which may result in new chronic kidney disease or advancement of pre-existing renal dysfunction [3, 4]. Additionally, since the mean age of onset is 65 years [1], many patients have coexisting medical issues that preclude them from major surgery. Presently, these patients have limited curative treatment options. Non-surgical treatment options for this population of patients include radiofrequency ablation (RFA) and cryotherapy. These thermal techniques have significant limitations. They can typically only treat smaller tumours which need to be away from the collecting system and vascular structures due to the risk of heat sink effects, stricture and/or fistula development [5]. Treatment of larger tumours poses significant risks of haemorrhage, which can require a nephrectomy to control [5]. Both RFA and cryotherapy are costly and dependent on user technician proficiency and availability. Both are invasive, with access through incisions in the skin. Thus, patients with inoperable primary RCC, particularly those with larger tumours, represent a group in need of an effective non-invasive alternative to surgery.

Stereotactic ablative body radiotherapy (SABR) is emerging as a non-invasive method for precision irradiation of tumours using doses with a higher biological effect than can be achieved with conventional radiotherapy. SABR does not necessitate inpatient hospital admission, is delivered in a single or relatively few treatment sessions, and is typically associated with a low toxicity and excellent local control rates in a variety of malignancies [6]. This radioablative technique represents a radical departure from conventional fractionated radiotherapy that is delivered with many treatments (fractions) over several weeks. SABR offers patients who are unable or unwilling to undergo surgery an effective alternative which can potentially prolong survival. SABR is now well established and widely utilised in the treatment of tumours of the brain, lung, liver, and spinal column [7–10].

In order to test this potential benefit of this technique in a larger randomised study, the initial step is to robustly establish efficacy of SABR for primary RCC. This multicentre phase II trial aims to validate the efficacy readout previously established by a pilot study conducted in the Australian setting [11].

### Existing knowledge

RCC is generally considered “radioresistant” in the context of conventionally fractionated radiotherapy. However, the capacity to deliver ablative doses of radiation accurately with SABR has shifted this paradigm [12]. The Karolinksa Institute in Sweden have the first reported clinical cases utilising SABR in the treatment of renal tumours, having treated kidney targets since the early 1990’s [13]. Wersall et al. [35] reported a retrospective series of 50 patients with metastatic renal cancer and 8 patients with inoperable primary tumours treated with SABR. Using a dose fractionation of 8Gy × 4, 10Gy × 4 and 15Gy × 3 they achieved a local control rate of 90–98%. 39% of patients developed at least one side effect, with 50% of registered side effects being grade I-II. In 2012 our group published a systematic review of 126 patients indicating favourable early results from SABR for primary RCC, with local control rates ranging from 84 to 100% and severe treatment related toxicities (grade 3+) of 3.8% [14]. Modern single institutional prospective reports from our centre [15, 16] and collaborators [17–19] corroborate these findings with very infrequent severe treatment related toxicities (< 5%). These studies report local control rates of 87% in 15 patients [19], 95% in 19 patients [17] and 100% in 30 RCC patients [18]. These findings are summarised in Table 1. A further recent study addressed the role of SABR in large primary RCC (those with a median tumour diameter of 9.5 cm) [20]. With a median follow up 3.9 years, there was one treatment related grade 3 event but no grade 4 or 5 toxicities. Importantly, in these studies, unlike RFA and cryotherapy, SABR was not limited to smaller tumours (< 4 cm) or those located away from central pelvic calyceal structures. Finally, the International Radiosurgery Oncology Consortium for the Kidney (IROCK) [21] published pooled results of 223 patients in 2018, with a 2-year local control rate of 97.8% [22].

**Table 1:** Review of SABR literature for primary RCC

Author / Year	Patients	Follow-up (months - Median or Mean)	Average marginal dose (Gy)	Outcome - Crude Local control	Estimated 2 year LC	Overall Survival Median	Toxicities
Chang et al. [7] 2016	16	19	30-40Gy in 5 fractions	100%	NR	NR	1 grade 2 acute toxicity and 2 grade 4 late toxicities
Gilson et al. [8] 2006	33	17	Median 40Gy in 5 fractions	94%	92	NR	NR
Lo et al. [9] 2014	3	21.7	40Gy in 5 fractions	100%	NR	NR	Early: 1 x Grade 1 nausea Late: nil
McBride et al. [10] 2013 (abstract) [prospective]	15	36.7	Median 33Gy in 3 fractions	87% 1 failure at 30.7 mo 1 failure at 31.2 mo	NR	NR	1 x Grade 3 renal toxicity 5 x Grade 1 fatigue 2 x Grade 1 nausea
Nair et al. [27] 2013	3	13.3	39Gy in 3 fractions	100%	NR	NR	Early: 1 x Grade 1 nausea Late: Nil
Nomiya et al. [28] 2008	10	57.5	Median 4.5Gy x 16fx	100%	100	5 year OS 74%	10% Grade 4 toxicity, no other toxicities > Grade 1
Qian et al. [29] 2003 (abstract)	20	12	40Gy in 5 fractions	93%	86	NR	NR
Pham et al. [15] 2014 [prospective]	20	6	26Gy in 1 fraction 42Gy in 3 fractions	NR	NR	NR	60% Grade 1–2 No Grade 3, Grade 4
Ponksy et al. [30] 2015 [prospective]	19	13.7	Max 48Gy in 4 fractions	NR	NR	NR	5.2% Grade 2, 15.8% Grade 3–4
Siva et al. [16] [prospective]	33	24	26Gy in 1 fraction 42Gy in 3 fractions	97%	100%	2 year OS 92%	78% Grade 1–2 3% Grade 3
Svedman et al. [31] 2006 [prospective]	5	52	40Gy in 4 or 5 fractions, 45Gy in 3 fractions	80%	91	Median survival 32 months	89% Grade 1–2, 4% Grade 3
Svedman et al. [32] 2008	7	39	40Gy in 4 fractions	86%	91	NR	58% Grade 1–2, nil else
Teh et al. [33] 2007 (abstract)	2	9	24Gy–48Gy in 3–6 fx	100%	100	NR	NR
Staehler et al. [18] 2015 ^b^ [prospective]	30^a^	28.1	25Gy in 1 fraction	98% (at 9mo) ^b^	NR	Not attained after median 28.1 months^**+**^	13% Grade 1–2 nil else
Wang et al. [13] 2014	9	38.3	36-51Gy to 50% isodose line at 3-5Gy per fraction	5 year LC 43%	NR	5 year OS 35%	Early: 44% Grade 1 (GI, haem.) Late: 22% Grade 2 (GI)
Wersall et al. [34] 2005	8	37	40Gy in 4 or 5 fractions, 45Gy in 3 fractions	100%	100	Median survival 58+ months	20% Grade 1–2, 19% Grade 3, nil Grade 4+

^a^ report included an additional 15 patients with Transitional cell carcinoma;

^b^ pooled results with patients treated for TCC

*NR* not reported, *Gy* Gray

### Addressing an unmet clinical need

As RCC is a disease typically of an older population, in routine clinical practice some patients are not fit for surgery due to existing co-morbidities. These patients need an effective non-invasive alternative to surgery to control their kidney disease. A multicentre, multinational clinical trial is warranted to assess the efficacy of SABR as a treatment option for primary RCC in medically inoperable patients or those who decline surgery.

## Methods/design

### Study objectives and hypothesis

The primary objective of this study is to estimate the efficacy of SABR for primary RCC. The primary endpoint is freedom from local progression, as defined by lack of progression of the target lesion (primary RCC) as measured by RECIST criteria [66]. The primary hypothesis of this study is that freedom from local progression at 1-year post-treatment will be > 80%.

Key secondary endpoints include assessment of a) Toxicity, as measured by CTCAE v4.03; b) Overall survival; c) Cancer specific survival; d) Freedom from distant failure; e) Renal function measured by serum creatinine and estimated glomerular filtration rate (eGFR) using CKI-EPI equation, split function and calculated glomerular filtration rate (GFR) on nuclear medicine testing; f) Cost-effectiveness. An exploratory objective is to assess the utility of multi-parametric MRI (mpMRI) as a biomarker of treatment response in a subset of patients.

This trial will be conducted in radiation oncology treatment centres throughout Australia, the MAASTRO clinic in the Netherlands, and the Royal Marden Hospital in England. All participating centres must successfully complete pre-trial quality assurance procedures prior to enrolling patients to the study. This will consist of an initial credentialing phase involving phantom dosimetry audit, facility questionnaires and a plan quality benchmarking exercise. Real-time quality assurance through plan reviews will be performed as part of the trial.

### Study design

The Trans-Tasman Radiation Oncology Group (TROG) 15.03 FASTRACK II clinical trial is a single arm, multi-institutional phase II study conducted in collaboration with the Australian and New Zealand Urogenital and Prostate Cancer Trials Group (ANZUP). The trial will recruit 70 participants over a 3-year period, with patient follow-up to 5 years from the end of accrual. All participants will receive SABR as definitive treatment for their primary RCC.

The study population is patients with biopsy confirmed primary RCC with a single lesion within a kidney. The intervention is a single fraction SABR with one of two fractionation schedules selected based on tumour size: a single fraction of 26Gy is used for tumours ≤4 cm in maximum diameter, with 42Gy in three fractions delivered for tumours > 4 cm in maximum diameter. While biological equivalent dose estimation based on linear quadratic formula can be unreliable to estimate the effect of doses >10Gy per fraction, this model is still the most commonly used to describe iso-effectiveness of alternate SABR fractionation regimens. The radiosensitivity of the two most common human RCC cell lines (Caki-1 and A498) has been observed in the laboratory to vary with α/β values estimated to be 2.6Gy or 6.9Gy, depending on the cell line [23]. The biological effective dose for tumour effects when using the dose/fractionation cohorts proposed with an α/β of 6.9Gy are 123Gy for 26Gy in 1 fraction, and 127Gy for 42Gy in 3 fractions. When altering the model to use the alternate α/β of 2.6Gy, the biological equivalent dose is 286Gy for 26Gy in 1 fraction and 268Gy for 42Gy in 3 fractions. These dose calculations show that the cohorts are relatively iso-effective across a reasonable range for potential tumour effects in the study.

### Inclusion / exclusion criteria

To participate in the trial, patients must meet all of the following key inclusion criteria at randomisation:

- Age ≥ 18 years old
- Biopsy confirmed diagnosis of RCC with no more than a single lesion within a kidney (bilateral RCC is allowable ECOG performance ≤2
- Life expectancy > 9 months
- Either medically inoperable, technically high risk for surgery or decline surgery
- Provide written informed consent
- A multidisciplinary decision has been made that active treatment is warranted

Key exclusion criteria are listed below:

- Pre-treatment estimated glomerular filtration rate < 30 mls/min
- Prior systemic therapies for RCC
- Previous high-dose radiotherapy to an overlapping region (defined as BED >40Gy using an α/β ratio of 10)
- Tumours larger than 10 cm in maximum dimension
- Direct contact of the target tumour with bowel
- Untreated prior malignancy, or prior malignancy within 2 years of screening
- Visceral / bony metastatic disease
- Horseshoe kidney

### Sample size and study duration

In total, 70 patients are required for this study. It is anticipated that it will take 36 months to complete target accrual. The sample size of 70 patients was chosen to provide sufficiently narrow confidence intervals for clinical outcomes. If up to 15% of participants do not proceed to treatment, or die or are lost to follow-up before experiencing a local progression in the first year, and assuming the freedom from local progression (FFLP) at 1-year is 90%, the corresponding two-sided 95% confidence interval will be 79–96%. A 1-year freedom from local progression of 80% or less will be considered undesirable and not worthy of proceeding to a future randomised study against other treatment modalities. The freedom from local progression at 1-year is expected to be around 90%. With those assumptions, the proposed sample size will have 80% power with 10% alpha to test the null hypothesis that the FFLP at 1-year is 80% or less. The study end date is deemed to be the date of last data capture, which will be when the final participant completes the 5 year follow-up visit. Participant assessments are tabulated in Table 2.

**Table 2:** Participant Assessments

ASSESSMENTS	Pre-Registration^a,b^	Post Treatment^c^													Post Progression^f^
		4 wks	3 mths	6 mths	9 mths	12 mths	18 mths	24 mths	33 mths	42 mths	51 mths	60 mths	Annually thereafter^d^	Progression^e^	Annually
Informed Consent	✓														
Informed consent for Health Economics data^g^	✓														
CT (Thorax/Abdomen)	✓^h,i^			✓	✓	✓	✓	✓	✓	✓	✓	✓	✓	✓^h,i^	
Split Renal Function Test (DMSA SPECT/CT) and calculated GFR (Cr-51 EDTA) ^j^	✓					✓		✓		✓		✓	✓^j^		
Whole body bone scan	✓													✓	
Clinical Consultation	✓		✓	✓	✓	✓	✓	✓	✓	✓	✓	✓	✓	✓	
Eligibility confirmation	✓														
Blood Tests^k^ and eGFR ^l^	✓	✓	✓	✓	✓	✓	✓	✓	✓	✓	✓	✓	✓	✓	
QLQ-C30 questionnaire ^m^	✓	✓	✓	✓	✓	✓	✓	✓	✓	✓	✓	✓			✓^m^
Adverse Event Reporting	✓	✓	✓	✓	✓	✓	✓	✓	✓	✓	✓	✓	✓	✓	
Survival Status															✓
MRI Sub study^n^															
Informed Consent	✓														
Multiparametric MRI	✓		✓			✓									

^a^To be done within 8 weeks of registration

^b^Treatment to commence within 6 weeks of registration

^c^Calculated from the date of the last radiotherapy fraction given. Post treatment follow-up to continue until both local and distant progression has been recorded

^d^After participant has both local and distant progression documented, annual survival status will be collected

^e^To be done at both local and distant progression

^f^After participant has both local and distant progression documented, annual survival status will be collected until study closure

^g^Australian Participants only for consent to access outpatient medical and pharmaceutical services via the MBS and PBS

^h^With or without contrast

^i^CT to include pelvis at pre-registration & progression scans. The target lesion size determined from the pre-registration scan CT will dictate the fractionation schedule to be prescribed

^j-^ Where split function cannot be performed by DMSA SPECT/CT, a MAG-3 study should be performed

- Calculated GFR through Cr-51 EDTA measurement or equivalent should be performed at this time point whenever possible, if this study is available

- This should be the same study for all investigations time points

- After the 60 month post follow-up visit, these are to be performed biennially until study completion

^k^Blood test include - full blood count (FBE), serum urea and electrolytes (UEC’s), c-reactive protein (CRP) and estimated glomerular filtration rate (eGFR)

^l^Determined by the CKD-EPI equation

^m^QLQ-C30 questionnaire is to be completed post progression until participant reached 5 years post treatment

^n^Participating sites only

### Radiotherapy treatment regimen

All participants are to be adequately immobilised using, at a minimum, a half body vacuum immobilisation device. A 4DCT scan in treatment position is used to account for respiratory motion. Target volumes accounting for respiratory motion and setup uncertainty must be defined as below:

- Internal Target Volume (ITV) – The ITV must be contoured taking the total tumour excursion through respiration into account.
- Planning Target Volume (PTV) – ITV to PTV margins must take into consideration set-up uncertainties. A 5 mm expansion isotropic expansion from ITV to PTV must be used.

For fractionated treatment schedules, treatment fractions are expected to be delivered on non-consecutive days (approximately 48 h apart). All treatment should be completed within 3 weeks.

### Dose criteria

The investigational treatment will be prescribed to the covering isodose, ensuring that 99% of the PTV is covered by 100% of the dose (D99PTV = 100%). In the circumstance where doses to organs at risk (OAR) cannot be respected whilst achieving this level of coverage, an alternative prescription coverage of D95PTV = 100% is acceptable. The peak dose (DMax) should be ideally 125%, resulting in a normalised equivalent covering isodose of 80%. The acceptable isodose at the periphery is expected to be between 70 and 80%. Peak dose (Dmax) should not exceed 143%. OAR constraints are found in Table 3.

**Table 3:** Organ at Risk Constraints

Organ	Parameter	Dose / Fractionation	
		26Gy/1Fx	42Gy/3Fx
Spinal canal	Maximum dose	0.03 cc < 12Gy point dose	0.03 cc < 18Gy point dose
Skin (5 mm subcutis)	Maximum Dose (1.5 cc)	< 18Gy	< 30Gy
Small Bowel	Maximum Dose / Volume	0.03 cc <26Gy 5cc < 22.5Gy Aim for <13Gy to full circumference of small bowel loop	0.03 cc < 30Gy 30cc ≤ 12.5Gy
	Maximum dose covering full circumference of bowel wall	≤12.5Gy	≤22.5Gy
Large Bowel	Maximum Dose (1.5 cc)	ALARA, aim for <26Gy	ALARA, aim for <42Gy
Stomach	Maximum Dose Maximum Volume	1.5 cc < 15.4Gy 5 cc ≤ 22.5Gy	0.03 cc < 30 Gy 5 cc ≤ 22.5Gy
Liver	Mean dose, Maximum Volume	No constraint, but mean dose and dose to 700 cc to be documented	700 cc < 15Gy
Ipsilateral kidney minus ITV	Maximum Dose (1.5 cc), V10Gy	ALARA: Minimise volume of high dose regions (> 50% isodose)^a^	ALARA: Minimise volume of high dose regions (> 50% isodose)
Contralateral Kidney	V10Gy	≤33%	≤33%

^a^ Ipsilateral Kidney minus ITV should adhere to the ALARA principle. In particular, investigators should focus on minimising high dose regions (> 50% isodose) outside the ITV but within the ipsilateral kidney

### Treatment delivery

**Fig. 1:**
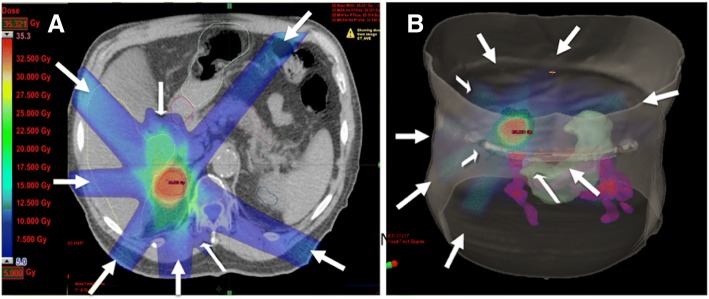
Axial 3D conformal treatment plan of SABR for right kidney (A) and 3D reconstruction (B) showing multiple beam angles resulting in high-doses wrapping tightly around the target

3D conformal treatment must be delivered with at least six non-opposing conformal megavoltage photon beams. It is anticipated that a typical range of beam numbers would be 8 to 12, comprising of at least 6 co-planar beams and 1–2 non-coplanar beams. For treatment with conformal or VMAT arc techniques a typical beam arrangement would include two 180–220 degree arcs avoiding entrance through the contralateral side. A representative treatment plan is demonstrated in Fig. 1.

### Treatment verification

All patients must undergo daily online image verification. Verification imaging must be capable of visualising the target with soft tissue matching as well as bone alignment. This would necessitate imaging with a Cone Beam CT (CBCT) or superior pre-treatment imaging modality.

### On-trial quality assurance (QA) program

This trial includes a comprehensive on-trial QA program. A pre-treatment radiotherapy QA technical review will be undertaken by the trial management team to provide independent validation of target definition, OAR contouring and plan quality. Post-treatment radiotherapy QA technical review will also be undertaken for all participating cases.

To ensure high plan quality and adherence to OAR dose limits, treatment plans will be assessed using knowledge based planning (KBP) prior to treatment. A RapidPlan model in the Eclipse treatment planning software (Varian Medical Systems, Palo Alto, USA) will be created based on the first 55 kidney SABR patients treated at Peter MacCallum Cancer Centre. The model will be applied to the first 20 patients recruited to the current trial to determine applicability to various planning systems and techniques used in this multi-centre trial. The model will then be used to determine if sufficient sparing of OARs has been achieved for each patient enrolled in the trial, based on previously achieved plan quality. This information will be communicated to the individual centres with the option to change the treatment plan prior to treatment if deemed appropriate given potential plan quality improvements and time and resource limitations.

### Adverse event reporting

All adverse events (AE) are to be reported between baseline and completion of study follow-up. All non-serious adverse events (including anticipated and unanticipated device related adverse events) shall be recorded in the trial database via adverse event CRFs. Internal statistical analysis of this data shall be performed at the times specified in the protocol and investigators and responsible HRECs will be advised of any safety issues which emerge during this process. Serious adverse event (SAEs) are to be reported to the Trial Coordinating Centre by the trial site within 24 h of being identified, from the commencement of radiotherapy to 90 days after the last fraction of radiotherapy. The investigator is responsible for notifying the responsible HRECs and Research Governance Officers as per local ethical and regulatory guidelines.

### Imaging sub-study 1: Evaluation of renal function change in response to SABR

Preservation of renal function is a major objective of kidney SABR. We will obtain split function nuclear medicine studies at baseline and 12, 24, 42 and 60 months after SABR for each patient enrolled in this trial. We will use deformable image registration to register the CT component of each DMSA SPECT/CT with the treatment planning CT, and apply this registration to the SPECT data. This will result in a 3D map of the DSMA uptake in the same spatial frame of reference as the planned radiotherapy dose. Based on this, we will then determine the change in DSMA uptake with radiation dose, and link any change as observed on imaging with clinical toxicity. Understanding the renal toxicity dose response will inform dose constraints on future patients. This information can further be used to optimise treatment plan quality in future renal SABR patients to minimise the dose to the functional components of the kidney.

### Imaging sub-study 2: Evaluation of renal function change in response to SABR

Multiparametric MRI (mpMRI) including diffusion weighted imaging (DWI) and dynamic contrast enhanced (DCE) MRI will be acquired in a subset of patients before treatment (baseline) and at 3 months and 12 months after SABR. DWI will be obtained using an echo-planar imaging sequence in the coronal plane with b-values of 50, 400 and 800 mm^2^/s, with the use of respiratory gating to reduce the effect of motion. DCE MRI will be obtained using a 3D T1-weighted TWIST sequence, in the coronal plane at an oblique angle so the entire affected kidney and the aorta are within the field of view. DCE MRI will be acquired during free breathing and 3D motion correction algorithms will be applied afterwards to reduce the effect of motion. Apparent diffusion coefficient (ADC) maps will be computed from the DWI data, and semi-quantitative and pharmacokinetic maps computed from DCE MRI data. Tumour contours will be drawn on the ADC maps and DCE MRI data, and relevant parameter statistics extracted. The change in each MRI parameter will be computed relative to baseline MRI, and correlated with the changes in tumour volume shown on follow-up CT relative to baseline CT, to assess whether DWI or DCE MRI parameters indicate treatment response earlier than conventional morphological CT based measures given by RECIST criteria [24] as shown in our exploratory study [25].

### Health economics evaluation

An economic evaluation will be conducted to estimate the incremental cost-effectiveness of SABR compared with current therapies (RFA/cryotherapy/surgery) for the treatment of patients with RCC. In the first instance, this will assess the within study costs and outcomes associated over the 5 years of the study. Subsequently, a Markov model will be developed using data on SABR from FASTRACK II and historical data on the comparators to assess the incremental cost-effectiveness of SABR compared with current therapies for the treatment of inoperable RCC.

The outcomes of the economic evaluation will be presented as cost per additional quality adjusted life year (QALY) gained. For SABR, QALYs will be estimated by applying utility values derived from the QLQ-C30 [26] by the time spent in each health state (e.g. progression free, progressed) and summed to calculate the total number of QALYs associated with SABR throughout the defined time-horizon. Utility values for the comparator technologies will be sought from the literature. In the absence of published values for those technologies, it will be assumed that there is no difference in utility values between therapies for a given health state.

Costs will be estimated taking into account the direct health care resources used for the delivery of SABR, associated hospital visits and the use of other medical services. Australian patients will be consented for access to administrative claims data from Medicare in order to capture outpatient medical and pharmaceutical services. Indirect patient costs (e.g. travel time and clinic time away from usual activities) will be assessed via a patient completed questionnaire, to be administered at the time of completing HRQoL questionnaires. Costs for the comparators will be based on existing scheduled fees and hospital costs known to apply to current therapies, supplemented with information on outpatient service use as informed by the data from this study.

Resource use will be valued using hospital-specific costs and scheduled fees for medical services and procedures. Mean estimates of costs will be used and Bayesian credibility intervals (BCI) will be generated by boot-strapping the data. The robustness and validity of the cost-effectiveness analysis will be explored using both a deterministic one-way sensitivity analysis and a probabilistic sensitivity analysis.

### Statistical considerations

Baseline characteristics will be summarised using descriptive statistics including counts and percentages for categorical variables and mean, standard deviation (SD), median and range for continuous variables. The Kaplan-Meier method will be used to estimate the OS, FFLP and freedom from distant progression. Annual estimates will be provided alongside 95% CI. An exploratory analysis will be carried out assessing the impact of baseline and treatment characteristic on OS using a Cox proportional hazard model. Cumulative incidence curves for cancer related death will be provided assuming competing risks with non-cancer related death as a competing event. Cumulative incidence curve for Grade 3+ toxicity will be provided assuming competing risks with death not related to treatment considered as a competing risk. A table with the frequency of worst AE grade experienced will also be provided for each individual AE type.

Change over time in renal function (serum creatinine, eGFR using CKI-EPI equation, split renal function and calculated GFR using nuclear medicine assessments) will be described using linear mixed models. The linear mixed model will include time as fixed effect (as a factor) and patients as a random effect. Mean and 95% confidence intervals will be calculated for each time point and the data will be displayed graphically using contrast from the linear mixed models. Changes from baseline will also be estimated using contrast from the linear mixed model. The impact of medical factors on renal function decline will be assessed by including relevant variables as independent variables in the linear mixed model.

No imputation of missing values and no adjustments for multiplicity are intended. This trial aims to a) describe clinical outcomes of patients with RCC treated with SABR and b) assess whether it is worthwhile conducting a future large randomised trial against other treatment modalities. The descriptive part of the study will use 5% alpha to provide two-sided 95% confidence intervals for clinical outcomes while the hypothesis testing on the FFLP at 1 year will use a one sided test with 10% alpha.

## Discussion

There is growing evidence to support the safety of SABR/SRS, its effect on local control and possible impact on overall survival for primary RCC. FASTRACK II is a study that will provide multicentre validation of the efficacy of SABR, which has previously only been demonstrated in single institutional and retrospective reports. At the time of writing, the study is open at eight sites across Australia and is actively recruiting, with two international sites in the process of activation. TROG 15.03 FASTRACK II will provide robust data on the efficacy of SABR for primary RCC in inoperable patients, to enable future larger phase III clinical trials.

## Abbreviations

4D: Four-dimensional
BED: Biological effective dose
CBCT: Cone beam CT
CT: Computerised tomography
CTCAE: Common terminology criteria for adverse events
DCE: Dynamic enhanced contrast
DMax: Peak dose
DWI: Diffusion weighted imaging
ECOG: Eastern co-operative oncology group
eGFR: Estimated glomerular filtration rate
FBE: Full blood count
HREC: Human research ethics committee
HRQOL: Health-related quality of life
ITV: Internal target volume
MRI: Magnetic resonance imaging
OAR: Organ at Risk
QA: Quality assurance
QALY: Quality-adjusted life year
RCC: Renal cell carcinoma
RFA: Radiofrequency ablation
SABR: Stereotactic ablative body radiotherapy
SRS: Stereotactic body radiosurgery
TCC: Transitional cell carcinoma
TROG: Trans tasman radiation oncology group
UEC’s: Serum urea and electrolytes
